# The Aggregation of Huntingtin and **α**-Synuclein

**DOI:** 10.1155/2012/606172

**Published:** 2012-07-26

**Authors:** María Elena Chánez-Cárdenas, Edgar Vázquez-Contreras

**Affiliations:** ^1^Laboratorio de Patología Vascular Cerebral, Instituto Nacional de Neurología y Neurocirugía, Insurgentes sur 3877 Col. la Fama, 14269 Mexico, DF, Mexico; ^2^Departamento de Ciencias Naturales, CNI, Universidad Autónoma Metropolitana Cuajimalpa, Pedro Antonio de los Santos 84 Col. Sn. Miguel Chapultepec Deleg, Miguel Hidalgo, 11851 México, DF, Mexico

## Abstract

Huntington's and Parkinson's diseases are neurodegenerative disorders associated with unusual protein interactions. Although the origin and evolution of these diseases are completely different, characteristic deposits of protein aggregates (huntingtin and *α*-synuclein resp.), are a common feature in both diseases. After these observations, many studies are performed with both proteins. Some of them try to understand the nature and driving forces of the aggregation process; others try to find a correlation between the genetic and failure in protein function. Finally with the combination of both approaches, it was proposed that possible strategies deal with pathologic aggregation. Unfortunately, if protein aggregation is a cause or a consequence of the neurodegeneration observed in these pathologies, it is still debatable. This paper describes the process of aggregation of two proteins: huntingtin and **α** synuclein. The characteristics of the aggregation reaction of these proteins have been followed with novel methods both *in vivo* and *in vitro*; these studies include both the combination with other proteins and the presence of various chemical compounds. The ultimate goal of this study was to summarize recent findings on protein aggregation and its possible role as a therapeutic target in neurodegenerative diseases and their role in biomaterial science.

## 1. Introduction

At present, it is evident that if proteins adopt their correct three-dimensional structure, that is, acquire their native structure; they will be able to perform multiple biological functions including macromolecular recognition, catalysis, and interaction with biomolecular targets among other possibilities. This conclusion was reached after studying protein denaturation, where the loss of the different levels of protein structure can be followed by many biophysical techniques. In some cases, when recovery of the structure and biological function of proteins were studied *in vitro*, it was observed that the process of nonspecific aggregation appears frequently; this situation is obtained using several types of proteins and perturbing with many denaturing conditions [1]. At the end of the twentieth century protein aggregation was observed *in vivo* in some pathological conditions. It was found that several phenomena may be involved in protein aggregation process. For example, some thermodynamic or kinetic situations could lead to the stabilization and association of partially folded intermediates [2], and it has been observed that some denaturing conditions induce the presence of nonnative conformations [3]. *In vivo*, the accumulation of nonfunctional proteins or their incorrect compartmentalization, is related to a failure to remove misfolded or nonfolded proteins, and with the disruption in the posttranslational control (inhibition of glycosylation or the reduction of disulfide linkages, among others). Also a genetic origin can be related with aggregation; on the one hand, it was found that mutations alter the protein stability, and secondly there are mutations that modify the rate of interconversion between the native state and the aggregate.

The reported pathological conditions may be the result of a loss of the original protein function, or by the gain of some toxic function. For a few human alterations such as cystic fibrosis, sickle cell anemia, and familial pulmonary emphysema, there is a clear relationship between an error in the protein folding and a cellular dysfunction. Moreover, there are cases where the proteins do not work or are not secreted in suitable amounts [4]. There are also a large number of pathologies, under the name of amyloidosis, in which the errors of protein folding lead to the irreversible formation of insoluble fibrillar aggregates called amyloids. This group includes several diseases, for example Alzheimer's disease, Creutzfeldt-Jakob disease, diabetes type II, and bovine spongiform encephalopathy. Currently, it is not known whether the presence of amyloid fibers in these pathologies is the cause or effect of cellular dysfunction. In fact, the causes of the formation of these fibrils are also debatable. Even though Huntington's (HD) and Parkinson's diseases (PD) have not been considered as amyloidoses, they involve the presence of a distinctive protein prone to aggregate. The protein aggregation associated with these disorders will be reviewed in the following sections.

## 2. Aims and Scope of the Chapter

Huntington and Parkinson diseases (HD and PD resp.) are two major neurodegenerative disorders pathologically characterized by the accumulation of the aggregate-prone proteins: mutant huntingtin (Htt) in HD and *α*-synuclein (*α*-syn) in PD. HD is the most common of nine inherited neurological disorders caused by expanded polyglutamine (polyQ) sequences, which confer propensity to self-aggregate and toxicity to their corresponding mutant proteins. On the other hand, *α*-syn is likely to be a major contributor to PD since overexpression of this protein from genetic triplication is sufficient to cause human forms of PD. Due to the importance of these diseases, researches around the Htt and *α*-syn aggregation are numerous. On the one hand, there are studies related to thermodynamic factors that govern the processes of stabilization of certain conformers that are involved in oligomerization; on the other hand, there is the study of the rate at which energy barriers are overcome to produce the three-dimensional species involved in the formation of the aggregates. Thermodynamic and kinetic processes are studied primarily *in vitro*; however, many experiments are carried out *in vivo*, using all the models and techniques available since cell lines to transgenic mice. Resent research has found that could be a relationship between the aggregation of these two proteins *in vivo*. To try to understand which are the molecular processes involved in the aggregation of these proteins, molecular biology is a very useful tool for dissecting the involvement of certain genes and the modification of its resulting proteins. Pharmacology is involved in the generation of molecules that could eventually be used as a probe, as well as inhibitors for the formation of the aggregates. Even it should be noted that all these molecular studies are related to research at the metabolic, cytological, and behavioral level because of the significance of the implications that these diseases have on the quality of human life. Also, some research in materials science have focused on the formation of aggregates of *α*-syn and HTT, as the seeds used to try to produce biomaterials. In this review, is described the proteins Htt and *α*-syn in HD and PD, respectively, as an approach to understand these pathologies as protein aggregation alterations. *In vivo* and *in vitro* aggregation studies of these proteins are reviewed in order to summarize recent findings on protein aggregation and its possible role as a target in neurodegenerative diseases.

## 3. Huntington's Disease (HD) and Huntingtin Protein

HD is an autosomal dominant neurodegenerative disease characterized by movement disorders and cognitive impairment. Psychiatric manifestations such as depression, irritability, and dementia are also observed. Brains with HD show a massive loss of striatum neurons (especially the medium-sized spiny neurons), although in advanced stages, the hippocampus, hypothalamus, cerebellum, amygdala, and thalamic nuclei are also affected. The symptoms of HD appear in midlife; however, it can range from childhood up to 70 years of age and over. In general patients die 10 to 15 years after the onset of symptoms, and no treatment or cure exists to date. 

In HD a mutation occurs in the exon 1 of the huntingtin gene (*HTT*). It results in an expanded allele with a variable length of repetitive trinucleotides “CAG.” In wild-type alleles, the length of CAG trinucleotides is between 6 and 37 and produces a 350 kDa protein-denominated huntingtin (Htt); on the other hand, in expanded alleles, the length of CAG trinucleotides ranges from 35 to 121 and produces proteins with a polyglutamine (polyQ) tract, variable in length. This characteristic includes HD in the family of polyQ diseases. Notably, the severity of the disease and the earlier age of onset are correlated with the length of the polyQ tract.

In humans Htt is a ubiquitously expressed protein; however, its precise role in metabolism is unclear, even so the importance of this protein is relevant because Htt knockout mice show cell degeneration and embryonic lethality [5]. It has been proposed that Htt is involved in different processes including neurogenesis, apoptosis, vesicle trafficking, caspases activation, signaling pathways, proteasomal function, and gene expression. Some consequences of Htt mutation have been reported; between them it is the loss of regulation of transcription of several kind of genes, for example, those involved with encoding neurotransmitter receptors, stress response, and enzymes and proteins involved in neuron structure. It was also reported a significant interaction of this protein with basal transcription factors such as TBP (TATA binding transcription factor), the transcription factor II F, and the cyclic AMP-response element binding (CREB) protein (CBP) amongst others (reviewed in [6]). It is known that transcriptional alterations are involved in HD and that the mutation of Htt could be part of this deregulation. Chromatin immunoprecipitation experiments have demonstrated that mutant Htt modifies the normal expression of specific mRNA species and then the gene expression is modulated through abnormal interactions of this protein with genomic DNA. Finally this interaction modifies the DNA conformation, producing alterations in the binding of transcription factors that changes the expression of those proteins [7]. 

Between the most relevant regulations produced by Htt is that of the brain-derived neurotrophic factor (BDNF). Cortical neurons produce this prosurvival factor that is necessary for the maintenance of striatal neurons in the brain. In the wild type cells Htt upregulates the transcription of BDNF; on the other hand in mutant Htt cells, the production of cortical BDNF decreases, then the neurotrophic support for striatal neurons is insufficient, causing cell death [8]. Mutant Htt affects not only BDNF transcription but it is known that wild-type Htt also promotes BDNF vesicular transport along microtubules, interacting with Htt-associated protein-1 (HAP1) and the p150 (Glued) subunit of dynactin, an essential component in molecular motors. The deficient mutant Htt transport of BDNF results in the loss of neurotrophic support and neuronal toxicity [9]. Finally it was found that the presence of Htt mutant protein diminishes the serum levels of BDNF in HD patients [10]. The participation of Htt in axonal transport, vesicle secretion, and trafficking has been studied using fusion proteins as the markers for fast axonal transport, and it was studied in presymptomatic homozygous mutant mice carrying 150 Q Htt knock-in mutations. It was found that the absence of Htt function also disrupts axonal transport, whereas the overexpression of the wild type (wt) but not of the mutant Htt enhances it [11]. On the other hand, the role of Htt in post-Golgi trafficking of secreted proteins was recently described. It was shown that mutant Htt perturbs post-Golgi trafficking to lysosomal compartments by delocalizing the optineurin/Rab8 complex, which in turn, affects the lysosomal function [12]. It was reported that mutant Htt has also been involved in excitotoxicity; the presence of this specie produces an abnormal increase of receptors for excitatory amino acids, especially those for N-methyl-D-aspartic acid (NMDA). Finally, observations concerning mutant Htt interactions with scaffold protein PSD 95 and transgenic mice overexpressing human exon 1 of Htt, show that the trafficking of NMDA receptor to the cell surface alters the presence of this receptor at the membrane level which in turn causes hypersensitivity [13, 14].

## 4. Huntingtin Aggregation

*In vivo* and in cell cultures it was observed that mutant Htt forms intranuclear inclusions bodies and cytoplasmic aggregates in neurons. Those forms are composed by N-terminal Htt fragments, which have been located as both neuronal intranuclear inclusions and dystrophic neurites in the cortex and striatum of HD patients [15]. It was found that the distribution of Htt inclusions is different throughout the different types of neurons; for example, two days after an adenovirus infection, it was observed that cytoplasmic inclusions become dominant in cortical and striatal neurons, whereas the opposite occurs on day 4 of the infection; that is, the ratio of nuclear inclusions overtakes that of cytoplasmic inclusions [16]. PolyQ proteins produce spherical and annular oligomeric structures; it is believed that they are reminiscent forms of those constituted by amyloid *β* in Alzheimer's disease and *α*-syn in PD. These annular structures have been observed in aggregates of polyQ containing proteins and other proteins associated with diseases of the brain. They have also been observed in large systems of polyQ peptide model simulations at a molecular-level [17]. Htt aggregates are colocalized with several proteins including those involved in proteolysis, transcription regulation, vesicle trafficking, and ubiquitin-proteasome system components (proteasome subunits and chaperones). It has also been suggested that these proteins are sequestered by mutant Htt, contributing to the pathogenesis of the disease [18], and that the cleavage of Htt by caspases and calpains plays an important role in the existence of inclusion bodies and aggregates. The cleavage of Htt for caspase 3 produces N-terminal fragments of the polyQ tract; consequently, the increase in these fragments promotes the formation of intranuclear inclusions; it was also observed that the inhibition of caspase activity reduces their formation. In order to understand the progression and pathogenesis of HD, several metabolic factors have been studied; it was found that both the proteolytic processing of the mutant Htt and the abnormal calcium signaling could play a critical role and that the mutant Htt is also a substrate of the calcium-dependent proteases and calpains. Several references indicate that calpains may participate in the increased and/or altered patterns of Htt proteolysis, leading to the selective toxicity observed in the HD striatum. It was reported that the mutation of two calpain cleavage sites in Htt produces a polyQ-expanded Htt, and this protein is less susceptible to proteolysis and aggregation than the wt forms, and produces a decreased toxicity in a cell-culture model [19]. Moreover, it has been suggested that not all Htt proteolytic fragments contribute to toxicity and therefore pathogenic as well as a nonpathogenic role has been suggested for the Htt cleavage by different caspases; for example, using mass spectrometry and site-directed mutagenesis, it has been recently demonstrated that the generation of a cp-2 fragment (specifically cleaved at the level of arginine 167) produces a pathogenic fragment; this study reveals that specific sites of Htt are involved in the toxicity produced by the mutant Htt in HD [20].

## 5. The Effect of PolyQ Length and Its Concentration in Htt Aggregation

From some observations, it may be argued that Htt aggregation has a pathogenic role in HD. The characterization of aggregates by agarose gel electrophoresis has shown that cytoplasmic (but not nuclear) Htt aggregates become larger as the disease progresses in the brain of transgenic mice; it has demonstrated the formation of these aggregates in primary striatal neurons and in the brains of R6/2 and Htt Q150 mice; it was also observed that the presence of aggregates preceded the initiation of any other functional deficits in the cell [21]. On the other hand, when purified Htt fragments were used to follow the Htt aggregation *in vitro*, it was observed by electron and atomic force microscopy that globular and protofibrilar states appear as intermediates in the mature of fibril aggregation process. This observation supports a strong correlation between structural changes of mutant Htt and the consequential appearance of mature fibrils and neurotoxicity. The observation of similar intermediates in aggregates such as amyloid *β* and *α*-syn, suggests that the presence of these conformers plays an important role in the neurotoxicity observed in HD and other neurodegenerative diseases [22]. Moreover, when a set of Htt with various glutamine repetitions (between 23 and 150 units) is expressed in cell culture, the aggregation of the protein was not induced when the repetition was ≤49 units; however, the process was promoted by repetitions ≥120 units, suggesting that for the reaction of aggregation the length of the polyQ tail is important [23]. This observation correlates with the HD age of onset, the longer the CAG expansion in the *HTT* gene, the earlier the HD symptoms appear. Furthermore, it was reported that Htt, with polyQ tracts in a pathological range (>37 Q), form sodium dodecyl sulfate (SDS) resistant aggregates with fibrilar morphology, while wt Htt does not. In fact, the study of coaggregation of mutant and wt Htt fragments showed that mutant Htt promotes the aggregation of wt aggregates with the characteristic fibrilar morphology; then it was proposed that this observation could be related with the loss of normal functions in neuronal cells of HD patients [24]. The formation of Htt aggregates not only depends on polyQ repetition length but also on protein concentration and time. For example, when the aggregation process is followed in transfected COS cells as a function of time, it was found that the formation of Htt aggregates follows a kinetic mechanism, in which nucleation is the rate-limiting step [25]. It was proposed that sufficiently long polyQ peptides first undergo a unimolecular conformational change, in which they form a nucleus that eventually seeds aggregation. This proposed nucleation mechanism has been used to derive a stochastic mathematical model to describe the probability of aggregate formation in cells, as a function of time and mutant Htt protein concentration [26]. The development of this kind of models has important implications in the design of therapeutic strategies for HD since it has been shown that modest reductions in Htt expression delay the aggregate formation. Theoretical simulations of Htt protein aggregation have confirmed important characteristics involved in the process, for example, the differential hydrophobicity between glutamine versus nonglutamine segments (which was originally proposed as the driving force of the association process), the protein concentration, and the length of the polyQ tract. These studies also demonstrate that the physicochemical behavior of the exon 1 fragment not only depends on the polyQ properties, but also on the rest of the sequence [27].

In Htt the C terminus of the polyQ (aggregation domain) could be flanked by polyproline (polyP) tracts regions; it was proposed that polyP regions could be “protective” against aggregation and cytotoxicity. For example, it was synthesized polyQ peptides with 3–15 glutamine residues and a corresponding set of polyQ peptides flanked on the C terminus by 11 proline residues (poly(Q)-poly(P)), as occurs in the Htt sequence [28]; it was observed that the shorter soluble polyQ peptides (three or six glutamine residues) present both a polyproline type II-like-(PPII) helix conformation (obtained by circular dichroism spectroscopy) and a monomeric organization (verified by size-exclusion chromatography); on the other hand, the longer poly(Q) peptides (nine or fifteen glutamine residues) showed a beta-sheet conformation and defined oligomers.

## 6. Proteasomal Dysfunction and Chaperones in Htt Aggregation

The generation of Htt aggregates is strongly determined proteasomal Htt degradation. Several studies show that in the presence of mutant Htt protein the accurate proteasome function is disrupted, increasing the level of Htt fragments and facilitating the formation of inclusions. As mentioned before, in these inclusions of Htt, chaperones and proteasomal subunits are “sequestered.” A few components of the ubiquitin system have shown to be potential modulators of Htt aggregation, and it was suggested that failures in ubiquitination could play an important role in the pathology of polyglutamine diseases. On the other hand, as mutant Htt forms insoluble aggregates associated with components of the ubiquitin system, including ubiquitin, ubiquitin-like proteins, and proteins that bind ubiquitin, it has been demonstrated that isolated ubiquitin-interacting motifs might serve as potential inhibitors of polyQ aggregation *in vivo *[29]. Besides, it was reported that the expression of single and isolated ubiquitin-interacting motifs inhibited aggregation of the mutant Htt. There is also information that E2-25K (Hip2), an ubiquitin-conjugating enzyme, which interacts directly with Htt, may mediate the ubiquitination of the neuronal intranuclear inclusions of Htt and that is involved in the aggregate formation, modulating aggregation, and toxicity of the expanded Htt [30]. It was also reported that several situations produces an increase in the aggregation of mutant Htt. For example a decrease in the proteasome activity as result of cellular conditions that promote proteasomal malfunction such as oxidative stress [31] and the presence of specific compounds [32] was observed.

It was proposed that the aminoterminal segment of the protein could be involved in the accumulation and aggregation process; in order to study this possibility, mutant Htt fragments (smaller than the first 508 amino acids) were generated in *HTT*-transfected cells and *HTT* knock in mouse brains. It was found that these fragments constituted neuronal nuclear inclusions, which appeared before the neurological symptoms. It was also observed that their accumulation and aggregation were associated with an age-dependent decrease in proteasome activity and that these process were promoted by the inhibition of proteasome activity. After these facts it was suggested that the decrease in proteasome activity contributes to a late onset of Htt toxicity and ultimately restore the ability to remove NH_2_-terminal fragments. These observations suggest a route to find a more effective therapy for HD rather than only the inhibition of their production [33].

On the other hand, a relevant role for chaperones in HD aggregation has been proposed. It has been shown that the aggregation of proteins with polyQ is not a consequence of proteolytic failure of the 20S proteasome. Rather and independent of proteolysis, aggregation is elicited by chaperone subunits of the 19S particle. Apparently, polyQ aggregation requires the transition between a benign conformation and an aggregation-prone form; it was proposed that proteasomal chaperones could facilitate the presence of this conformation [34]. Moreover, it has been reported that two molecular chaperones (HSP70 and HSP40) modulate the polyglutamine aggregation reactions by partitioning monomeric conformations and disfavoring the accretion of spherical and annular oligomers [35]. For example, *in vitro* and in an ATP-dependent process, the presence of these chaperones produces the suppression of the assembly of Htt into detergent-insoluble amyloid-like fibrils and, however, caused the formation of amorphous, detergent-soluble aggregates. Besides, it was observed that these chaperones were most active in preventing fibrillization when added during the lag phase of the polymerization reaction. Finally, when the system was repeated in yeast cells, similar inhibition detergent-insoluble polyQ aggregates were observed [36].

## 7. Chaperon-Like Proteins That Decrease Htt Aggregation

Recently, the use of novel chaperones, nonmammal chaperones, and proteins with chaperon-like activity has shown that it can be useful in the decrease of Htt aggregation. For example, the chaperonin TRiC, TCP-1 Ring Complex, also called CCT for chaperonin containing TCP-1, is a cytosolic protein that can alter the course of aggregation and cytotoxicity of Htt in mammalian cells. The aggregation states of the Htt in the presence and absence of TRiC were analyzed by fluorescence correlation spectroscopy. These studies revealed that the depletion of TRiC results in the appearance of soluble Htt aggregates. It was found that the eukaryotic chaperonin is composed of eight different subunits, each thought to be represented once per eight-membered ring. It was found that the overexpression of all eight subunits of CCT suppressed Htt aggregation and neuronal cell death [37]. The chaperone p97 plays a protective role in neurodegenerative disorders suppressing the protein aggregation. It was found that, in *Caenorhabditis elegans*, two homologues of chaperone p97, CDC-49.1 and CDC-48.2, partially suppress aggregation of the *HTT* exon1 fragment. It has been proved that CDC-48.1 and CDC-48.2 bind to the *HTT* exon1 fragment directly, suppressing the formation of SDS-insoluble aggregates of Htt fragments and also modulating the oligomeric states during the aggregate formation [38]. Recently, it has been reported that Htt interacts with the cue domain of gp78 and inhibits gp78 binding to ubiquitin and p97 [39]. Htt-interacting protein (HYPK) alters the number and distribution of aggregates formed by N-terminal Htt with 40 Q in Neuro2A cells. Even though HYPK does not possess any sequence similarity with the known chaperones it was found that it also modify the kinetics of mutated N-terminal Htt-mediated aggregate formation. Also it was reported that both* in vitro* and *in vivo* HYPK possesses a novel chaperone-like activity; then it was suggested that this protein play an effective role in protecting neuronal cells against apoptosis induced by mutated N-terminal of Htt; it was proposed that this process occurs by modulating the formation of aggregates [40]. Moreover, HYPK acts together with NatA protein (N(alpha)-terminal-acetyltransferase complex) in cotranslational N-terminal acetylation and prevention of Htt aggregation [41]. Finally it was found that the yeast chaperone HSP104 reduces the aggregation of polyglutamine protein. This observation is supported by both the generation of a new transgenic mouse, which overexpresses the yeast chaperone HSP104, as well as the result of crossing of *HSP104* transgenic mice with mutant mice that express only the first 171 residues of mutant Htt. The overexpression of HSP104 reduces the aggregation process and prolongs the survival of a transgenic mouse model of HD. Then it was proposed that the reduction and protection processes are apparently due to a change in the conformation of a putative toxic monomer of the protein, reducing oligomerization or aggregation or the combination of both possibilities [42]. 

## 8. Could Aggregation in HD Be a Therapeutic Target?

Preventing the formation of insoluble Htt aggregates in neurons may represent an attractive therapeutic strategy to ameliorate HD symptoms. Different strategies to decrease Htt aggregation have been developed, such as the use of antibodies, oligonucleotides, and different compounds that modify Htt fibrillogenesis. The advance of novel methods for testing new drugs that are able to reduce the aggregation and toxicity of Htt has become a relevant issue; for example, the fluorescence resonance energy transfer-(FRET) based screening assay is a recent improvement in the screening of libraries of small active biological molecules, even in mammalian cells and *Drosophila* model. Recently, the use of these models has been found to be very effective to test drugs against Htt aggregation, providing new therapeutic tools for HD [43]. Other methods, such as the automated filter retardation assay, have been used for the identification of chemical compounds, which prevent the aggregation of *HTT* exon 1 product *in vitro*. This method was used in the screening of molecules that suppress the self-assembly of Htt, in order to find compounds with clinical and research applications. For example, twenty-five benzothiazol derivatives, which inhibit Htt fibrillogenesis in a dose-dependent manner, were discovered in a library of approximately 184,000 small molecules. Furthermore, cell culture studies revealed that 2-amino-4,7-dimethyl-benzothiazol-6-ol, a chemical compound similar to riluzol, significantly inhibits the aggregation *in vivo* of the *HTT* exon 1 product [44]. Other compound, the rapamycin, an immunosuppressant drug used to prevent rejection in organ transplantation, also reduces the amount of soluble polyQ protein via the inhibition of protein synthesis, which in turn significantly reduces the formation of insoluble polyQ protein and the formation of inclusion bodies. Therefore, a modest reduction in Htt synthesis by rapamycin may lead to a substantial decrease in probability of reaching the critical concentration required for a nucleation event and the subsequent toxic polyQ aggregation [45]. The use of compounds such as rapamycin has been proposed as promising therapeutic tools. For example, *in vitro* Htt studies have shown that reaching a critical concentration of these molecules is crucial for a nucleation event; only after this concentration is reached, the aggregation of toxic polyQ proceeds. In mammalian cells other compounds, such as pharmacologically improved derivatives of geldanamycin (17-DMAG and 17-AAG), have been proved to inhibit protein aggregation apparently through the induction of heat-shock response. It was observed that 17-DMAG induces the expression of HSP40, HSP70, and HSP105 chaperones. It was also found that this molecule inhibits the formation of mutant Htt aggregates with a greater efficiency than 17-AAG or even geldanamycin [46]. Efforts to prevent the misfolding and aggregation of Htt also involve the use of intracellular antibodies against Htt. It was observed that high levels of intrabody expression have been required to obtain even a limited number of reductions in aggregation. On the other hand, it was found that engineered single-domain intracellular antibody against Htt inhibits aggregation at low expression levels; it is believed that this process occurs by increasing its affinity in the absence of a disulfide bond [47]. Since the rate limiting of the nucleation process of polyQ aggregation involves the folding of mutated Htt monomers, a monoclonal antibody which selectively recognizes expanded pathogenic and aggregate-prone polyQ repetitions has been used. A direct correlation between polyQ tract aggregation, lag times and the intensity of the anti-1C2 signal in soluble monomers of Htt was found [48]. Finally, following the same logic, the use of short synthetic oligonucleotides has also been proposed as a therapeutic approach in blocking Htt nucleation and elongation phases of aggregation. It was found that introducing modified, single-stranded oligonucleotides into the neuronal cell line, the inclusion formation, is retarded [49]. In addition, it was shown that short guanosine single-stranded DNA oligonucleotides are capable to adopt a G-quartet structure and then be effective inhibitors of aggregation; this inhibition was tested in a cell-based assay and revealed that this oligomer is an effective inhibitor of the aggregation of both, the mutant Htt fragments and a green fluorescent protein fusion [49].

## 9. ***α***-Synuclein in Parkinson's Disease (PD)

PD is the second most frequent progressive neurodegenerative disorder. Clinical manifestations of PD include motor disorders such as a resting tremor, rigidity, and bradykinesia (abnormally slow movements). Pathologically, PD is characterized by the progressive and selective degeneration of the dopaminergic neurons in the *substantia nigra (SN)*. A variety of cellular and biochemical alterations are observed in PD: (1) Mitochondrial dysfunction caused by complex I inhibition; (2) excitotoxicity; (3) oxidative stress and subsequently oxidative damage to protein, lipid, and nucleic acid molecules; (4) inflammatory response by microglia activation and a raise in cytokine levels; (5) proteaseome dysfunction and (6) proapoptotic and autophagic pathways. In addition, it has been demonstrated that the neurodegeneration of the *SN pars compacta *is accompanied by protein aggregation in the Lewy bodies; these proteinaceous inclusions are localized in the cytoplasm and axon of the dopaminergic neurons of the *SN* although other areas are also affected. Sporadic or idiopathic PD accounts for a large number of cases. However, <5–10% of them are considered to be familiar PD, with a genetic component. The genes associated to familial forms of PD mediate autosomal dominant or recessive forms and include *α*-syn (SNCA, also known as PARK1), Ubiquitin C-terminal hydrolase L1 (UCH-L1, also known as PARK5), Leucine-rich repetition kinase 2 (LRRK2, also known as PARK8), Parkin (PARKIN, also known as PARK2), DJ-1 (also known as PARK7), and PTEN-induced kinase 1 (PINK1, also known as PARK6). The study of familiar PD genes and their mutation is useful to understand the underlying mechanisms of the disease. Protein aggregation is a key factor in the pathogenesis of both the familial and sporadic disease. The Lewy bodies are the pathological hallmarks of PD and they appear in both PD forms. Sporadic PD shows evidence of extensive protein damage. However, it has been proposed that the impaired degradation of these misfolded proteins is a consequence of a pathological process such as oxidative stress, mitochondrial as well as proteasomal dysfunction, inflammation, and excitotoxicity. In familial PD on the other hand, related mutations of *α*-synuclein (*α*-syn), as well as the duplication or triplication of the gene correlate with the possibility of the misfolding and aggregation of this protein. These observations suggest that the overexpression of *α*-syn is responsible of aggregation. This section of the text is focused on the protein product of the *PARK1* gene, the *α*-syn protein, due to its unfolded nature and the increasing interest in the folding studies and its proposed role as a therapeutic target for PD. However, it is interesting to note that the other familial PD genes (*PARKIN*, *UCHL-1*, *DJ-1*, *PINK1,* and *DARDARIN/LRRK2*) are also related with the cellular unfolding protein response. PARKIN is an E-3 ubiquitin ligase, which labels proteins with ubiquitin, tagging them for their degradation in the proteasome. Mutations in the Parkin gene lead to a deficiency in ubiquitination. UCH-L1 is a deubiquitination enzyme, and its function is to cleave ubiquitin. Defects in this protein could impair proteasomal clearance of misfolded proteins and reduce the availability of ubiquitin monomers. DARDARIN/LRRK2 mutations suggest that it promotes abnormal phosphorylation and aggregation of target proteins [50]. The involvement of DJ-1 and PINK-1 in protein aggregation is not clear; however, it has been observed that mutations in DJ-1 could promote damage to the free-radical-mediated protein and PINK-1 mutations could reduce the ability to protect cells from proteasome inhibition. Recently, it has been reported that PINK-1 exists as a dimer in mitochondrial protein complexes that comigrate with respiratory chain complexes in sucrose gradients. In cell culture systems, mutant PINK-1 or PINK-1 knockdown caused deficits in mitochondrial respiration and ATP synthesis. Furthermore, proteasome function is impaired with the loss of PINK-1, and deficits are accompanied by an increase in *α*-syn aggregation [51]. On the other hand, DJ-1 shows antioxidant and chaperone-like activities. With the development of a cellular model of oxidative stress to address the subject of whether *α*-syn and DJ-1 interact functionally, it has been demonstrated that the inactivation of DJ-1 may promote the aggregation of *α*-syn, also proving that HSP70 is involved in the antioxidant response and in the regulation of *α*-syn fibril formation [52]. Mutations in several of these familial PD genes have also been sporadically identified in PD, suggesting that their study might provide insight into both familial and sporadic forms. 

## 10. ***α***-Synuclein Aggregation

synuclein family is formed by the *α*-, *β*-, and *γ*-isoforms all of them are proteins between 15 and 20 kDa. *α*- and *β*-synuclein colocalize in presynaptic nerve terminals, close to synaptic vesicles, while *γ*-synuclein appears to be axonal and cytosolic. In addition to synucleins, a wide range of proteins, neurofilaments, and ubiquitin-proteasome system proteins are present in Lewy bodies. *α*-syn is the main component of these structures and could be present in its full-length, oligomeric form and in aggregates. It was also observed that these forms present several posttranslational modifications including phosphorylation, gycosylation, nitration, or ubiquitination [53]. As *α*-syn aggregates are present in PD, it was proposed that the study of the process could be an approach for therapeutics. For example, a new and useful tool for study aggregation is bimolecular fluorescence complementation, which allows for the direct visualization of *α*-syn oligomeric species in living cells, facilitating the study of initial events and leading to determine how manipulations affect the process [54]. 

*α*-syn is a monomeric protein considered to be “unstructured,” because of its lack of rigid structure. It is described as a protein with a remarkable conformational plasticity since it can be found in several forms including unfolded protein and amyloidogenic partially folded conformations [55]; it has also been reported that it can fold into *α*-helical or *β*-structure. In addition, *α*-syn can form different kind of aggregates, including oligomers, amorphous aggregates, or amyloid-like fibrils [56]. Experimental evidence has shown that the causes of *α*-syn assembly into fibers could involve several situations including an increase in concentration, inappropriate interaction with other proteins, posttranslational modifications (such as phosphorylation, partial or inappropriate cleavage of the flexible and highly negatively charged C terminus), and oxidative posttranslational modifications; eventually all of them result in the formation of oxidized and nitrated *α*-syn form [56]. Numerous efforts have been made to understand the early events of *α*-syn aggregation such as oligomerization and nucleation. For example, it has been reported that the photoinduced cross-linking of unmodified *α*-syn produces species with different oligomeric nature in solution, including monomers, dimers, and trimers [57]. It was proposed that it is probable that the seeds for *α*-syn aggregation are the dimeric and trimeric species, and that the N-terminal amphipathic region of the protein is necessary for the oligomerization process. Using fluorescence lifetime imaging, two forms of *α*-syn homomeric interactions have been detected: (1) an antiparallel interaction between the amino and carboxy terminus of *α*-syn molecules and (2) a close amino terminus-carboxy terminus interaction within single *α*-syn molecules [58]. The central hydrophobic region of *α*-syn is critical for the *β*-sheet interaction [59]. Furthermore, conformational heterogeneity of the monomeric *α*-syn has been characterized at a single-molecule level. It was found that, in this condition, there is equilibrium between a random coil, a mechanically weak fold, and *β*-like structures. It was also proposed that this conformational equilibrium controls the population of the specific class of monomeric *α*-syn conformers. Besides, these species are positively correlated with the conditions previously used to promote the formation of aggregates, such as the presence of Cu^2+^, the pathogenic A30P mutation, and high ionic strength [60]. Finally, PD-associated mutations in *α*-syn have been used to determine the presence of intermediates in the fibrillization process of the wt protein. For example results from fluorescence and CD experiments combined with singular value decomposition method demonstrate that wt *α*-syn presents a multistep process in the formation of fibrils. It was found that there are at least five intermediates present in the early stages of fibrillation [61]. Even though unstructured form of *α*-syn in solution is capable to form *β*-sheet-rich fibrils that accumulate as intracytoplasmic inclusions, a distinct protein conformation of the protein is observed when bound to phospholipid membranes. It was observed that in the presence of acidic phospholipid vesicles, the N terminus of *α*-syn folds into an amphipathic helical conformation that mediates membrane binding [62]. This fact suggests that lipid-dependent protein interactions might stabilize the folded structure of *α*-syn, and that this stabilization could decrease the oligomerization process. *In vivo*, *α*-syn is in dynamic equilibrium between membrane-bound form and soluble cytoplasmic specie. Therefore, understanding the role of lipid binding in modulating both the *α*-syn conformations and its aggregation is an important issue to propose PD therapeutics. In order to contribute to solve this questions, the thermodynamic characterization of the monomeric *α*-syn folding was performed. The experiments were carried out in the presence of SDS and followed by far-UV CD spectroscopy in order to detect the conformational transitions induced by the detergent, temperature, and pH. It was observed that the *α*-syn folding is a multistate process and that the structure of two of the intermediates stabilized is predominantly *α*-helical, however, they are only partially folded [63]. Moreover, to understand how the *α*-syn aggregation state affects its lipid binding activity, surface plasmon resonance, fluorescence anisotropy, and CD spectroscopy experiments were performed [64]. It was identified that during the early aggregation phase in the formation of fibrils, an intermediate with high affinity lipid binding appear [63]. On the other hand, to study the binding of *α*-syn to proteins in its lipid-associated helical conformation, a new *in vitro* approach was developed [65]. This procedure uses bacteriophage display to screen proteins that selectively bind to *α*-syn in this induced conformation. Interestingly, it was identified that 20 different human brain proteins specifically interact with phospholipid-bound *α*-syn; this observation confirms that the helical N terminus of *α*-syn can mediate specific interactions with other proteins and suggests that membrane binding may regulate their physiological activity *in vivo*. 

It was argued that oxidative stress has been implicated in a number of neurodegenerative disorders including PD. An increasing amount of evidence shows that the *α*-syn aggregation process can be altered by oxidative and nitrative damage. It is widely known that reactive oxygen species induce lipoperoxidation, and that one of the most important products of this reaction, 4-hydroxy-2-nonenal (HNE), is implicated in the pathogenesis of PD. In order to know if there is an interaction between HNE and *α*-syn, the protein was incubated in the presence of the compound [66]; it was found that the process produces covalent modifications of the protein, with up to six HNE molecules being attached. The characterization of the modified protein by Fourier transform, infrared and CD spectra, indicated that HNE modification of *α*-syn resulted in a major conformational change involving an increase in the *β*-sheet content. It was also observed that the HNE-modified *α*-syn does not undergo fibrillation and is argued that this is due to the formation of stable soluble oligomers in a HNE concentration-dependent manner. In addition, HNE-induced oligomers showed to be toxic for dopaminergic neurons in cell cultures [66].

## 11. ***α***-Synuclein Aggregation and Degradative Dopamine Metabolism

It has been proposed in the literature that the dopamine (DA) metabolism can be related with both *α*-syn aggregation and DA neuron loss in PD. Experiments *in vitro* demonstrated that the DA interaction with *α*-syn inhibits the fibril formation by stabilizing oligomeric kinetic intermediates [67]. Therefore, extensive research has been done in order to understand the effect of DA concentration, oxidation and metabolism. First, it was reported that the presence of DA causes the aggregation of the wt *α*-syn. On the other hand, it was observed that the A53T mutation in the *α*-syn gene might predispose the mutant protein to aggregation. Then it was found that the presence of DA causes the aggregation of both wt and mutated proteins in a temperature-dependent manner; however, the pathogenic A53T mutant shows a greater propensity to aggregate. It was suggested that this point mutation results in a structural change of the protein that drastically increases its propensity to aggregate in the presence of DA, contributing to PD pathogenesis [68]. Finally an *in vivo* cellular model, which regulates the intracellular steady-state levels of DA by expressing different forms of tyrosine hydroxylase, was developed in order to study aggregation of *α*-syn [69]. In these conditions, the formation of *α*-syn aggregates was inhibited and that the formation of innocuous oligomeric intermediates was induced. These results support the observations that intraneuronal DA levels can be a major modulator of the *α*-syn aggregation and the inclusion formation; both processes have important implications on the selective degeneration of these neurons in PD [70]. Moreover, the formation of *α*-syn aggregates is inhibited by oxidized catechols, stabilizing their soluble oligomeric intermediates. For example, it was reported that some of the oxidative modifications to *α*-syn are produced by the reactive orthoquinone and aminochrome species generated by catechols oxidation. To characterize the mechanism of DA-induced alterations in *α*-syn aggregation, the same cellular model that regulates DA concentration mentioned above was used [69]. In this condition it was found that the wt protein was modified in five residues; it was proposed that they compose a catechol interaction domain, responsible for inhibiting *α*-syn aggregation. Furthermore, it was demonstrated that DA and other catechols modulate the progression of PD through interactions with the C-terminal of *α*-syn [71]. It was also shown that aberrant C-terminal processing of *α*-syn might lead to selective vulnerability of dopaminergic neurons by accelerating the formation of insoluble inclusions. To understand the link between the *α*-syn aggregation and DA metabolism, the monoamine oxidase (MAO) metabolite of DA, 3,4-dihydroxyphenylacetaldehyde (DOPAL), was tested using several techniques including Western blot, fluorescent confocal microscopy, and immunohistochemistry in a cell-free system; the effect of DOPAL was tested *in vitro* (in DA neuron cultures) and *in vivo* (with stereotactic injections into the *SN* of Sprague-Dawley rats). It was found that in the cell-free system *in vitro* and in the cell cultures, the use of DOPAL in physiologically relevant concentrations triggers the formation of toxic *α*-syn oligomers that finally aggregate. On the other hand, the results in the *in vivo* system showed that the use of DOPAL resulted in DA neuron loss and the accumulation of high molecular weight oligomers of *α*-syn [72].

## 12. Therapeutic Research in ***α***-Synuclein Aggregation

As mentioned before, *α*-syn aggregation has become an important target in PD-therapeutic studies. Several strategies are developed in order to propose a treatment to prevent the aggregation of *α*-syn, including those that act directly with the protein and those that modify certain metabolic parameters that ultimately cause inhibition of the oligomerization. For example, it was proposed that some antibodies could inhibit the formation of the particularly toxic small oligomeric aggregates of *α*-syn; the dopamine (DA), nicotine, curcumin, and heat shock proteins as well as several NSAIDs regulate the *α*-syn fibril formation. On the other hand, it was found that oxidative stress and the presence of cholesterol results in mechanisms that can influence the formation of stable *α*-syn fibrils; the use of agonists and inhibitors for enzymes related with oxidative stress has been proposed as an antioxidant therapeutic strategy that could be involved in the inhibition of oligomeric forms of *α*-syn. Finally the regulation of cholesterol concentrations, with specific inhibitors as the statins, might be a novel approach for the treatment of PD. In order to find a therapeutic result against *α*-syn aggregation, several cell lines, mutant variants of the protein, and experimental approaches have been used. 

Since the DA-induced oxidative stress has been related with PD pathology, the use of DA agonists, as well as monoamine oxidase *β* inhibitors has been proposed as an antioxidant therapeutic strategy. The effect of anti-Parkinsonian agents was studied such as selegiline, DA, pergolide, bromocriptine, and trihexyphenidyl in the formation of *α*-syn fibrils and in preformed fibrils by several methodologies including the fluorescence of thioflavin S, electron microscopy, and atomic force microscopy experiments. The results demonstrate that in a dose-dependent response and except for trihexyphenidyl, all the molecules tested inhibited the formation of the fibrils and destabilized the preformed *α*-syn fibrils [73]. It was proposed that the use of antibodies to neutralize the particularly toxic small oligomeric aggregates of *α*-syn could be used in therapeutics against PD [74]. For example, a single-chain variable fragment (scFv) antibody against the nonamyloid component of *α*-syn was produced; the presence of this antibody reduces both the intracellular aggregation and the toxicity produced by the protein [75]. Other antibodies that recognize the different morphologies of *α*-syn have been obtained. For example using a phage-displayed antibody library, an antibody was found that binds to the oligomeric form of *α*-syn; it was shown that this *in vitro *antibody decreases both aggregation and toxicity [76]. Moreover, a second antibody, which binds to a larger but latter stage oligomeric form of *α*-syn was also reported; this antibody inhibits aggregation *in vitro*, blocking extracellular *α*-syn and inducing toxicity in both an undifferentiated and differentiated human neuroblastoma cell lines. Interestingly, it was found that this antibody specifically recognizes naturally occurring aggregates in PD, but not in healthy human brain tissue [77]. On the other hand it has been proposed that interactions among closely related homologous proteins, included in the same family, might regulate the state of aggregation in PD. For example, it is known that *α*-syn adopts *β*-sheet-rich conformations that promote the oligomerization and neurotoxicity; on the other hand, it has been observed that *β* synuclein lacks the central hydrophobic cluster observed in *α*-syn. This observation suggests that *β* synuclein could be a negative regulator for the amyloid formation of *α*-syn. In order to understand the molecular mechanism through which *β*-synuclein exerts its anti-amyloidogenic effect, the study of the structural and dynamic features of the protein has been performed characterizing the ensemble of *β*-synuclein conformations in a transgenic mice line [78]. It was observed that this model presents both a marked reduction in *α*-syn expression in the cortex and an overexpressing *β*-synuclein. This overexpression produces a reduction of *α*-syn protein expression; however it was not accompanied by a decrease in *α*-syn mRNA. In addition to this observation, retardation in the progression of impaired motor performance, reduction in aggregation of both *α*-syn (*α*-syn A53T mouse model) and *β*-synuclein was obtained, suggesting relevant implications of *β*-synuclein in the development of therapies for PD [79]. Moreover, the use of the cholesterol synthesis inhibitors (statins) lovastatin [80], simvastatin, and pravastatin was tested in *α*-syn aggregation in a transfected neuronal cell line as well as in primary human neurons. It was observed that the use of statins reduced the levels of *α*-syn accumulation in the detergent insoluble fraction of the transfected cells. This observation was accompanied by several effects including redistribution of *α*-syn in caveolar fractions, reduction in oxidized *α*-syn, and enhanced neurite outgrowth. In these experiments the addition of cholesterol to the media increased *α*-syn aggregation in detergent insoluble fractions of transfected cells. The cholesterol also reduces neurite outgrowth, suggesting that regulation of cholesterol levels, with specific inhibitors, might be a novel approach for the treatment of PD [81]. Based on epidemiological studies, it was proposed that the therapeutic use of nonsteroidal anti-inflammatory drugs (NSAIDs) could reduce the risk to develop PD. The effects of NSAIDs on the formation and destabilization of *α*-syn fibrils were studied using fluorescence spectroscopy in presence of thioflavin S and electron microscopy. The effect of several NSAIDs molecules including ibuprofen, aspirin, acetaminophen, meclofenamic acid sodium salt, sulindac sulfide, ketoprofen, flurbiprofen, diclofenac sodium salt, naproxen, and indomethacin, was tested. It was found that except for naproxen and indomethacin, the rest of the molecules inhibited the formation of *α*-syn fibrils in a dose-dependent response. It was proposed that NSAIDS also could destabilize preformed fibers [82]. In addition, it was reported that nicotine in a dose-dependent response inhibits the *α*-syn fibril formation in both wt and A53T forms. In addition, it was found that nicotine could destabilize preformed fibrillar *α*-syn in a dose-dependent manner, and that both effects could be attributed to the N-methylpyrrolidine moieties of the nicotine. These observations are a relevant issue in PD therapeutics since they correlate in retrospective and prospective epidemiological studies with an association between cigarette smoking and PD [83, 84]. Curcumin is a constituent of the Indian curry spice Turmeric, a herb of the ginger family, which is structurally similar to Congo red. It is known that curcumin is involved in the prevention of the oligomerization of A*β*-amyloid protein; considering this observation, the effect of this molecule in *α*-syn aggregation was tested. The addition of curcumin to an *in vitro* model of *α*-syn decreases the aggregation in a dose-dependent manner and increases its solubility. The aggregation-inhibiting effect of curcumin was investigated in the catecholaminergic SH-SY5Y cell line. Images captured using a high-throughput cell-based screening microscope showed that curcumin inhibits *α*-syn oligomerization into higher molecular weight aggregates. These observations suggest that curcumin could be a potential therapeutic compound for PD [85]. Finally, it was reported that some chaperones, specifically HSP70, are involved in regulate the *α*-syn fibril formation [52]. The assembly of *α*-syn in the presence of HSP70 was studied, and it was found that *in vitro* the assembly of *α*-syn was efficiently inhibited by substoichiometric concentrations of purified HSP70 in the absence of cofactors [86]. In fact, the use of *α*-syn deletion mutants indicated that interactions between the HSP70 substrate binding domain and the *α*-syn core hydrophobic region underlie assembly inhibition. Finally, it was found that the mammalian protein quality control system increases by the introduction of a nonmetazoan HSP104 in a rat lentiviral model of PD. This observation showed that both the dopaminergic neurodegeneration and the phosphorylated *α*-syn inclusion formation are reduced. HSP104 prevented fibrillization of *α*-syn and PD-linked variants (A30P, A53T, and E46K) and might have therapeutic potential in PD [87]. 

## 13. Cross Talk between ***α***-Synuclein and Huntingtin

After observing that HD and PD have characteristics in common, some research groups have studied the possible relationships between Htt and *α*-syn *in vivo*. For example, it was found that polyQ inclusions in HD and other polyQ disorders are immunopositive for *α*-syn; after this observation it was speculated that *α*-syn might be recruited as an additional mediator of polyQ toxicity; the hypothesis was confirmed because the accumulation of *α*-syn in polyQ inclusions was found in HD postmortem brains and in the R6/1 mouse model of HD. It was also found that N-terminal mutant huntingtin (N-mutHtt) and *α*-syn form independent filamentous microaggregates in R6/1 mouse brain as well as in the inducible HD94 mouse model and that N-mutHtt expression increases the load of *α*-syn filaments [88]. In the same direction, however, using a bimolecular fluorescence complementation assay, it was found that the initial steps of the coaggregation of Htt and *α*-syn it was found that HTT (exon 1) oligomerized with *α*-syn and sequestered it in the cytosol. In turn, *α*-syn increased the number of cells displaying aggregates, decreased the number of aggregates per cell, and increased the average size of the aggregates [88]. Finally, it was demonstrated that wild-type *α*-syn overexpression impairs macroautophagy in mammalian cells and in transgenic mice [89]. Then, and considering that the overexpression of human wild-type *α*-syn in cells and Drosophila models of HD worsens the disease phenotype it was examined whether *α*-syn overexpression also worsens the HD phenotype in a mammalian system using two widely used N-terminal HD mouse models (R6/1 and N171-82Q). The effects of *α*-syn deletion in the same N-terminal HD mouse models, as well as assessed the effects of *α*-syn deletion on macroautophagy in mouse brains were also studied and it was found that overexpression of wild-type *α*-syn in both mouse models of HD enhances the onset of tremors and has some influence on the rate of weight loss. On the other hand, *α*-syn deletion in both HD models increases autophagosome numbers, and this is associated with a delayed onset of tremors and weight loss, two of the most prominent endophenotypes of the HD-like disease in mice [90]. 

These observations therefore suggest for one hand that *α*-syn could be a modifier of polyQ toxicity *in vivo*, or in other words maybe in mammals there is a functional link between these two aggregate-prone proteins. On the other hand, these observations raise the possibility that potential PD-related therapies aimed to counteract *α*-syn toxicity might help to slow HD. Finally, these results support the idea that coaggregation of these aggregation-prone proteins can contribute to the histopathology of neurodegenerative disorders, helping with diagnosis.

## 14. Protein Aggregation and Biomaterials

Proteins are large, complex molecules, which play many critical roles in the body. They do work in cells and are essential for the structure, function, and regulation of the body's tissues and organs. In addition to their natural functions, proteins can also act as biocatalysts and biomaterials. A biomaterial is a synthetic or natural material used to replace part of a living system or to function in intimate contact with living tissue. The study of the biophysical properties of specific biomaterials constitutes a first approach and a critical role for applications in tissue engineering, reconstructive surgery and regenerative medicine. The design of biomaterials that elicit specific cellular behavior constitutes a major challenge for the fields of tissue engineering and materials science [91]. Therefore, the rational design technique is often used in order to produce biomaterials from proteins; this method functions through protein engineering and requires an understanding of the molecular interactions that stabilize proteins in specific folded configurations [92]. However, naturally programmed self-assembly proteins offer significant promise for the generation of new types of well-defined multifunctional materials to provide structural and functional supports. Anyway, the information on the biophysical properties of proteins, during and after their aggregation, can be used to manipulate the aggregates in different forms (gel, film, or solid matrix), depending on the final use and application of the new biomaterial. Collagen, fibrin, and albumin are popular proteins for making biological scaffolds for tissue engineering because of their biocompatibility, biodegradability, and availability. Considering that a major drawback of biological protein-based biomaterials is the limited control over their biodegradation, the properties of the naturally occurring aggregates have been studied. For example, the stability and robustness of amyloid-like fibrils, their nanoscale dimensions, their suitability for chemical modification, and their inherent biological compatibility, are generating increasing interest in the use of these fibrils as new biomaterials. In this sense, as *α*-syn turned into two morphologically distinctive amyloid fibrils—“curly” (CAF) versus “straight” (SAF)—depending on its fibrillation processes. The differences in secondary structures of CAF and SAF have been studied and suggested to be responsible for their morphological uniqueness with structural flexibility and mechanical strength. Accumulation of CAF produced amyloid hydrogel composed of fine nanoscaled three-dimensional protein fibrillar network resulting in a nanomatrix for enzyme entrapment; then nano-scaled fibrillar network of CAF is a potential application in the promising areas of nanobiotechnology including tissue engineering, drug delivery, nanofiltration and biosensor development [93]. Also a nanomaterial fabrication was performed utilizing two peptide fragments of key region for *α*-synuclein fibrillation. These peptides modify the fibril nanostructure of full-length *α*-synuclein, and the effects depend on the peptide sequences. It was proposed that the combination of amyloid-forming protein, and its partial peptide fragments with some mutations have a potential for novel nanomaterial fabrication [94]. Then, the cases of Htt and *α*-syn are studied in several ways that will open the access to the future understanding of these and other proteins that could be related with the generation of innovative materials with various purposes.
